# Identification and validation of ECM1 as a causal plasma biomarker in knee osteoarthritis through proteome-wide association study and bioinformatics

**DOI:** 10.1097/MD.0000000000043993

**Published:** 2025-08-22

**Authors:** Huadong Wang, Yinhui Yao, Xuelian Yin, Qingzhu Zhang

**Affiliations:** a Chengde Medical University, Chengde, China; b The Affiliated Hospital of Chengde Medical University, Chengde, China.

**Keywords:** Bayesian colocalization, bioinformatics, ECM1, knee osteoarthritis, proteome-wide Mendelian randomization

## Abstract

Knee osteoarthritis (KOA) is a degenerative joint disease with a genetic component. Nonetheless, it remains largely unknown how risk variants influence the OA risk through their effects on proteins. This study aimed to identify new and effective drug targets for the diagnosis of KOA. A proteome-wide association study was conducted using summary datasets from genome-wide association studies of OA and protein quantitative trait locus data. The Gene Expression Omnibus dataset GSE63359 was used to identify differentially expressed genes between controls and KOA. Subsequently, Mendelian randomization and colocalization analyses were performed on the intersecting proteins identified using the aforementioned methods to assess the association between protein levels and KOA risk. Clinical samples were included, and RT-qPCR and enzyme-linked immunosorbent assay were used to validate the expression of risk proteins between the KOA and control groups. Extracellular matrix protein 1 (ECM1) expression was causally related to KOA. Further support from colocalization analysis suggested that ECM1 might serve as a potential drug target for KOA. Validation of clinical samples indicated that ECM1 levels were higher in the KOA group than in the control group and that ECM1 demonstrated significant diagnostic efficacy for KOA (area under the curve = 0.85). This study identified ECM1 as a risk factor for the pathogenesis of KOA and showed promising therapeutic targets for KOA treatment.

## 1. Introduction

Osteoarthritis (OA), a prevalent chronic degenerative joint disease in the field of orthopedics, primarily affects the knee joint.^[1–5]^ Its clinical manifestations include pain, swelling, stiffness, and limited mobility of the knee joint. In 2019, healthcare expenditures for early-onset OA totaled $46.17 billion, with an additional $60.70 billion lost due to productivity.^[6]^ This global burden has led to disability and economic challenges.^[7–9]^ Approximately 500 million individuals worldwide are affected by OA, with knee osteoarthritis (KOA) accounting for more than 30% of the cases.^[10]^ Factors such as an aging population and the obesity epidemic contribute to the increasing prevalence of this condition.^[11–13]^ Therefore, early diagnosis and prevention of KOA are crucial. Nevertheless, the exact pathophysiological mechanisms underlying KOA remain incompletely understood, although genetic,^[14,15]^ environmental,^[16,17]^ and age-related factors have been suggested to play key roles.^[18,19]^ A deeper exploration of its pathogenesis can help guide the development of effective prevention and treatment strategies.

Genome-wide association studies (GWASs) utilize single-nucleotide polymorphisms (SNPs) as markers to identify genetic factors associated with complex diseases and provide insights into the genetic mechanisms underlying disease initiation, progression, and treatment.^[20–23]^ In recent years, GWASs have been conducted to uncover the genetic causes of OA, leading to the identification of several SNPs and genes associated with its pathogenesis.^[24–27]^ Examples include ALDH1A2, TGFB1, FGF18, CTSK, and IL11.^[24,28]^ However, because GWAS results are presented in the form of SNPs, understanding their impact on genes or proteins is challenging. To address this limitation, a novel analytical approach called “proteome-wide association study” (PWAS) has been developed to elucidate the roles of proteins in disease initiation and progression.^[29,30]^ PWAS has been performed for various diseases, such as venous thromboembolism, post-traumatic stress disorder, autism, migraine, and Parkinson disease, using tissue-specific proteins instead of blood proteins.^[29,31–40]^ Recently, the release of human plasma proteomic data has provided opportunities to explore the relationship between plasma proteins and OA risk.

In the present study, we combined data from GWAS and protein quantitative trait locus (pQTL) analyses to identify novel proteins that could serve as diagnostic and therapeutic targets in KOA. Additionally, we employed Mendelian randomization (MR) and Bayesian colocalization analyses to investigate the causal relationship between the identified proteins and KOA pathogenesis. The potential diagnostic value of candidate proteins was validated using Gene Expression Omnibus statistics. Finally, clinical samples were collected to verify the proteins associated with KOA.

## 2. Materials and methods

### 2.1. GWAS

GWAS summary statistics were generated from the Integrative Epidemiology Unit OpenGWAS project (https://gwas.mrcieu.ac.uk/) using GWAS ID ebi-a-GCST007092. The diseases covered in the GWAS summary statistics of this project included hip and knee OA. The total sample size was 48,108 participants, with 39,427 OA cases and 8681 healthy controls. A total of 30,265,359 SNPs were included in the dataset.

### 2.2. Proteomic data

Whole blood pQTL data were obtained from the Atherosclerosis Risk in Communities (ARIC) study (http://nilanjanchatterjeelab.org/pwas/),^[37]^ which included 1348 cis-heritable plasma proteins derived from 7213 European Americans to align with the GWAS datasets. Proteomic profiling was performed using SomaScan platform version 4.1 (SomaLogic, Inc., Boulder). Genotyping was conducted using the Infinium Multi-Ethnic Global BeadChip array (GenomeStudio, Illumina) and subsequently imputed to the TOPMed reference panel (Freeze 5 on GRCh38). These pQTL data were used as reference weights for the subsequent PWAS analysis.

### 2.3. PWAS

For PWAS analysis, GWAS summary statistics were integrated with the reference human plasma proteomes from the ARIC study^[37]^ using the Functional Summary-based Imputation (FUSION) pipeline (http://gusevlab.org/projects/fusion/). The KOA genetic effect (PWAS z-score) was calculated and combined with the precalculated plasma proteome reference weight (z-score × proteome weight) to evaluate the effects of significant SNPs in the GWAS on protein abundance. FUSION identified candidate genes associated with OA that regulate plasma protein abundance. To control for the potential effects of multiple testing on the study results, a Bonferroni correction was applied, establishing a significance threshold of *P* < 3.7 × 10^-5^ (0.05/1348) for our PWAS analysis.

### 2.4. Expression analysis of candidate genes

GSE63359 (https://www.ncbi.nlm.nih.gov/geo/) provided the protein expression data for KOA blood tissues. This dataset included 72 blood tissue samples from 46 Caucasian patients with KOA and 26 healthy controls. Differential analysis was performed on the normalized KOA statistics using the “limma” package to identify differentially expressed genes (DEGs), applying criteria of an adjusted *P*-value of < .05 and |log fold change| >0.25. The results obtained from the PWAS were compared with those of the DEGs to identify potential diagnostic markers for OA. The diagnostic performance of these markers was evaluated using receiver operating characteristic (ROC) curves.^[41]^

### 2.5. MR analysis

MR analysis is a genetic approach that uses genome-wide significant SNPs as instrumental variables (IVs) to explore causal relationships between exposure and outcome.^[42]^ A critical step in MR analysis is the selection of qualified IVs, which must satisfy 3 fundamental assumptions. First, the relevance assumption requires that genetic variants are directly associated with the exposure, typically determined by setting a significance threshold of *P* < 5E-08 to filter SNPs. Second, the independence assumption states that genetic variants are not directly related to confounding factors. Third, the exclusion assumption mandates that genetic variants do not directly influence the outcome.^[43]^

Qualified IVs were derived from variants that showed linkage disequilibrium with *r*² value < 0.001 and were located within 10 Mb. These IVs were extracted from the outcome traits and harmonized across the exposure and outcome GWAS datasets. If only 1 independent IV was available, the MR effect was estimated using the Wald ratio; if 2 or more IVs were available, the inverse variance-weighted method was employed.^[44,45]^ Statistical significance was set at *P* < .05. Moreover, when 3 or more IVs were available, sensitivity analyses including MR-Egger regression, weighted mode, weighted median mode, and simple mode analyses were conducted to ensure the robustness of the results. Subsequent MR analyses included the MR-Egger intercept test for pleiotropy assessment, Cochran *Q* test for heterogeneity evaluation, and the MR-PRESSO outlier test. However, after clustering, most pQTLs had only 1 or 2 IVs, rendering sensitivity and MR analyses infeasible. This algorithm was implemented using the “TwoSampleMR” R package (github.com/MRCIEU/TwoSampleMR).

### 2.6. Bayesian colocalization analysis

Colocalization (COLOC) analysis was conducted to determine whether 2 associated signals were consistent with shared causal variants.^[46]^ The default COLOC priors were used to test the posterior probabilities of 5 hypotheses: H0, no association with any trait; H1, association with trait 1, no association with trait 2; H2, association with trait 2, no association with trait 1; H3, association with both traits 1 and 2 but with 2 independent SNPs; and H4, association with both traits 1 and 2, indicating the existence of a shared SNP. A posterior probability for hypothesis 4 >0.8 was considered indicative of consistency between the 2 associated signals with shared causal variants. Regional association plots were generated using the “LocusCompareR” R package (https://github.com/boxiangliu/locuscomparer).

### 2.7. Blood sample collection

Data were collected from participants at the Affiliated Hospital of Chengde Medical University. The inclusion criteria for patients were based on the KOA diagnostic criteria established by the American College of Rheumatology in 2001. Patients with knee pain were required to meet at least 3 of the following 7 conditions: (a) age ≥ 50 years, (b) morning stiffness lasting for no more than 30 minutes, (c) crepitus during joint motion, (d) radiographic evidence of osteophytes, (e) bone tenderness, (f) absence of significant synovitis, and (g) radiological evidence of bone spur formation. The control group comprised healthy individuals who voluntarily participated in the study, excluding those diagnosed with KOA. The exclusion criteria were as follows: major illnesses that could affect essential clinical information relevant to the project’s examinations (such as malignant tumors, respiratory failure, and inability to walk normally), lack of blood biochemical tests, and unavailability of imaging data necessary for determining KOA.^[47]^ The X-ray equipment used was Ysio (Siemens, Germany). This study was approved by the Medical Ethics Committee of the Affiliated Hospital of Chengde Medical University (ethics number: CYFYLL2023222). Informed consent was obtained from all participants. All human trials adhered to the requirements of the Declaration of Helsinki (1964). Blood samples were collected from patients during fasting.

### 2.8. Verification of clinical samples

Total RNA was extracted using TRIzol immediately after blood collection, followed by cDNA synthesis using a reverse transcription kit. Specific primers for extracellular matrix protein 1 (ECM1) and GAPDH were designed by Sangon Biotechnology (Shanghai, China) (Table 1). The reaction mixture included cDNA, SYBR Green Master Mix, and the designed primers. Amplification was performed using the StepOnePlus real-time fluorescence quantitative PCR instrument (Heal Force, China) under the following conditions: an initial denaturation at 95 °C for 30 seconds, followed by 40 cycles of amplification (95 °C for 5 seconds and 60 °C for 30 seconds). Data analysis was conducted using the 2^-ΔΔCt^ method to quantify gene expression levels. Blood protein levels were measured using enzyme-linked immunosorbent assay (ELISA) following the manufacturer’s instructions (ELISA kit catalog number: ml803521S; Shanghai Enzyme-linked Biotechnology Co., Ltd.).

**Table 1:** Primer sequence used in RT-qPCR.

Gene	Primer sequence (5’–3’)
ECM1	F: GGACATCTTGAVATCGG R: TCACAGCAGCGGGCAGTC
GAPDH	F: GCACCGTCAAGGCTGAGAAC R: TGGTGAAGACGCCAGTGGA

### 2.9. Statistical analysis

Statistical analyses were conducted using R software version 4.2.2 (https://www.r-project.org/). Group comparisons were performed using the Wilcoxon test for skewed data and *t* test for normally distributed data. The ROC curve was plotted, and the area under the curve (AUC) value was calculated using the “ROCR” package (https://cran.r-project.org/web/packages/ROCR/index.html). Univariate and multivariate logistic regression analyses were performed using the “rms” package.^[48–51]^ Statistical significance was set at *P* < .05.

## 3. Results

### 3.1. PWAS analysis

**Figure 1.**
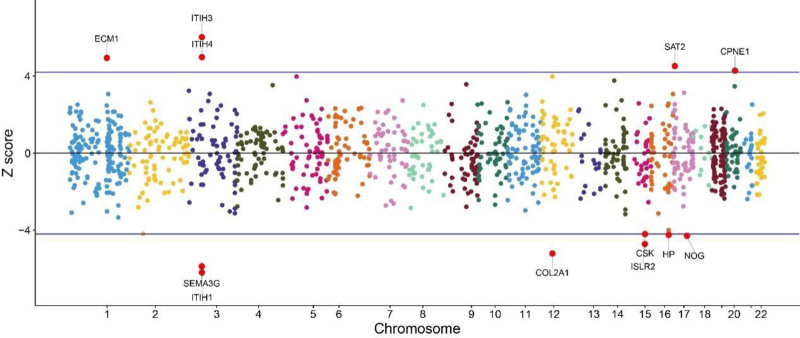
A Manhattan plot for the OA PWAS generated by merging the OA GWAS (N = 48108) with the ARIC proteomes (n = 1348). ARIC = Atherosclerosis Risk in Communities, GWAS = genome-wide association study, OA = osteoarthritis, PWAS = proteome-wide association study.

Based on the FUSION analysis of serum pQTLs involving 1348 proteins, 13 proteins were identified to be significantly associated with OA risk (*P* < 3.60 × 10⁻⁶). As detailed in Table 2, 5 of these proteins (namely, ITIH3, ITIH4, ECM1, SAT2, and CPNE1) had PWAS z-scores ˃0, indicating that they function as risk factors for OA. Conversely, the remaining 8 proteins (namely, ITIH1, SEMA3G, COL2A1, ISLR2, NOG, HP, CSK, and EFEMP1) had PWAS z-scores <0, suggesting that they act as protective factors against OA. Figure 1 presents a Manhattan plot of the PWAS results for OA, integrating findings from the OA GWAS (N = 48,108) and ARIC proteomic study (n = 1348).

**Table 2:** PWAS results for OA.

Protein	Chromosome	PWAS z-score	PWAS *P*-value
ITIH1	3	-6.19	5.73 × 10^-10^
ITIH3	3	6.01	1.81 × 10^-9^
SEMA3G	3	-5.87	4.15 × 10^-9^
COL2A1	12	-5.21	1.89 × 10^-7^
ITIH4	3	4.97	6.39 × 10^-7^
ECM1	1	4.93	7.82 × 10^-7^
ISLR2	15	-4.72	2.35 × 10^-6^
SAT2	17	4.51	6.29 × 10^-6^
NOG	17	-4.29	1.76 × 10^-5^
CPNE1	20	4.27	1.88 × 10^-5^
HP	16	-4.24	2.17 × 10^-5^
CSK	15	-4.20	2.63 × 10^-5^
EFEMP1	2	-4.19	2.78 × 10^-5^

OA = osteoarthritis, PWAS = proteome-wide association study.

### 3.2. Analysis results of DEGs

**Figure 2.**
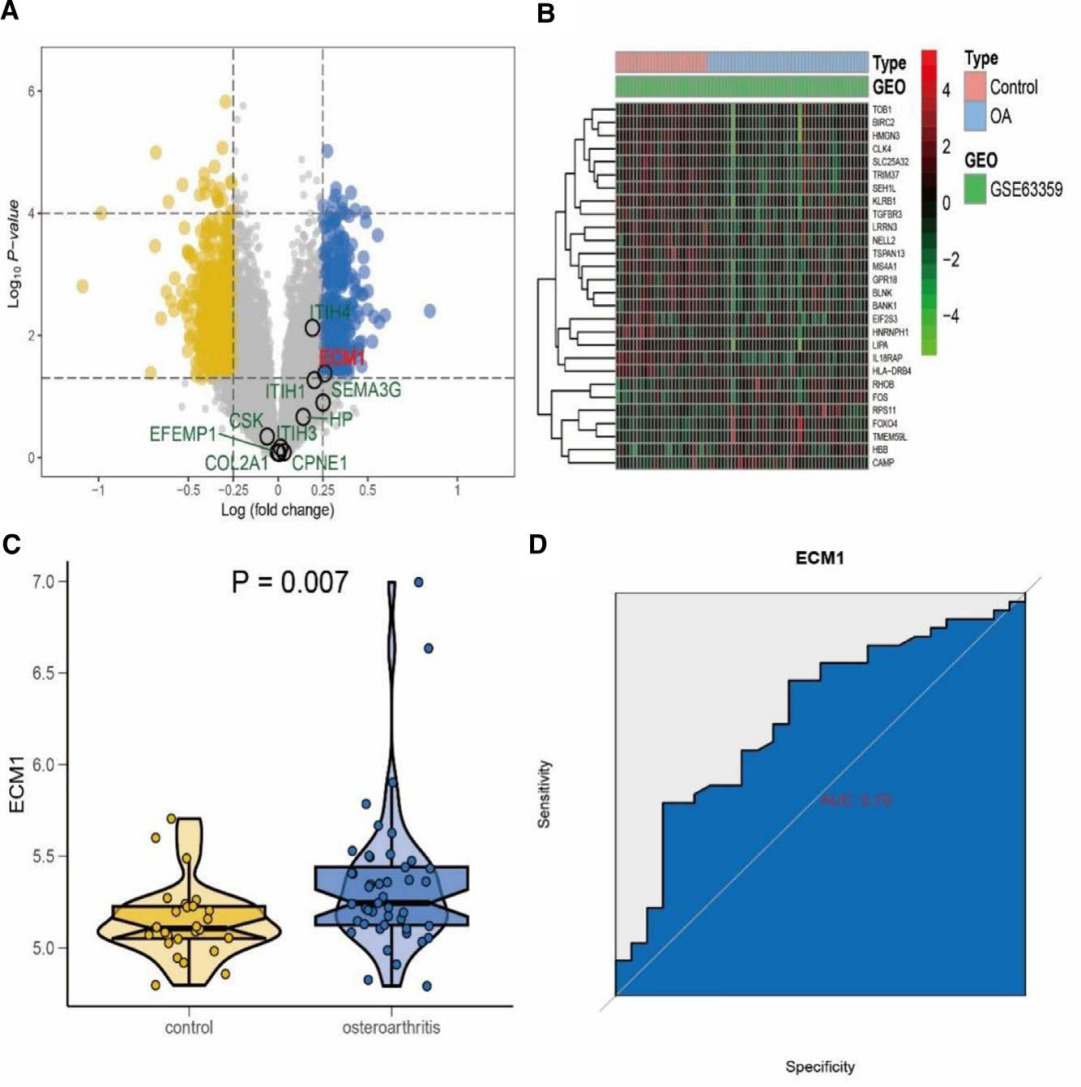
Screening and ROC curves of DEGs. (A) Volcano diagram of GSE63359. (B) Heat map of GSE63359. (C) Comparison of ECM1 in GSE63359 between OA and control groups. (D) ROC curve of ECM1 in GSE63359 for OA diagnosis. DEGs = differentially expressed genes, OA = osteoarthritis, ROC = receiver operating characteristic.

Based on the selection criteria, 855 DEGs were identified (Fig. 2A and B); among these, 321 were upregulated, whereas 534 were downregulated. The intersection of DEGs with PWAS revealed a single gene: ECM1. As shown in Figure 2C, ECM1 exhibited statistically significant differences between OA and normal controls in the GSE63359 dataset (*P* < .05). The AUC value for ECM1 was 0.7, indicating its potential diagnostic value in OA (Fig. 2D). Furthermore, the diagnostic indicator distinguished KOA-positive from KOA-negative cases.

### 3.3. MR analysis between proteins and OA

Preliminary screening of the exposure data yielded 1242 IVs. After several rounds of filtering, the OA outcome dataset identified the SNP rs11582094. The MR estimates were based primarily on the Wald ratio method. The results indicated that genetically predicted ECM1 might have a positive causal effect on OA, with an odds ratio of 1.06 (95% confidence interval: 1.01–1.11, *P* = .0054), which is statistically significant.

### 3.4. Bayesian colocalization analysis

**Figure 3.**
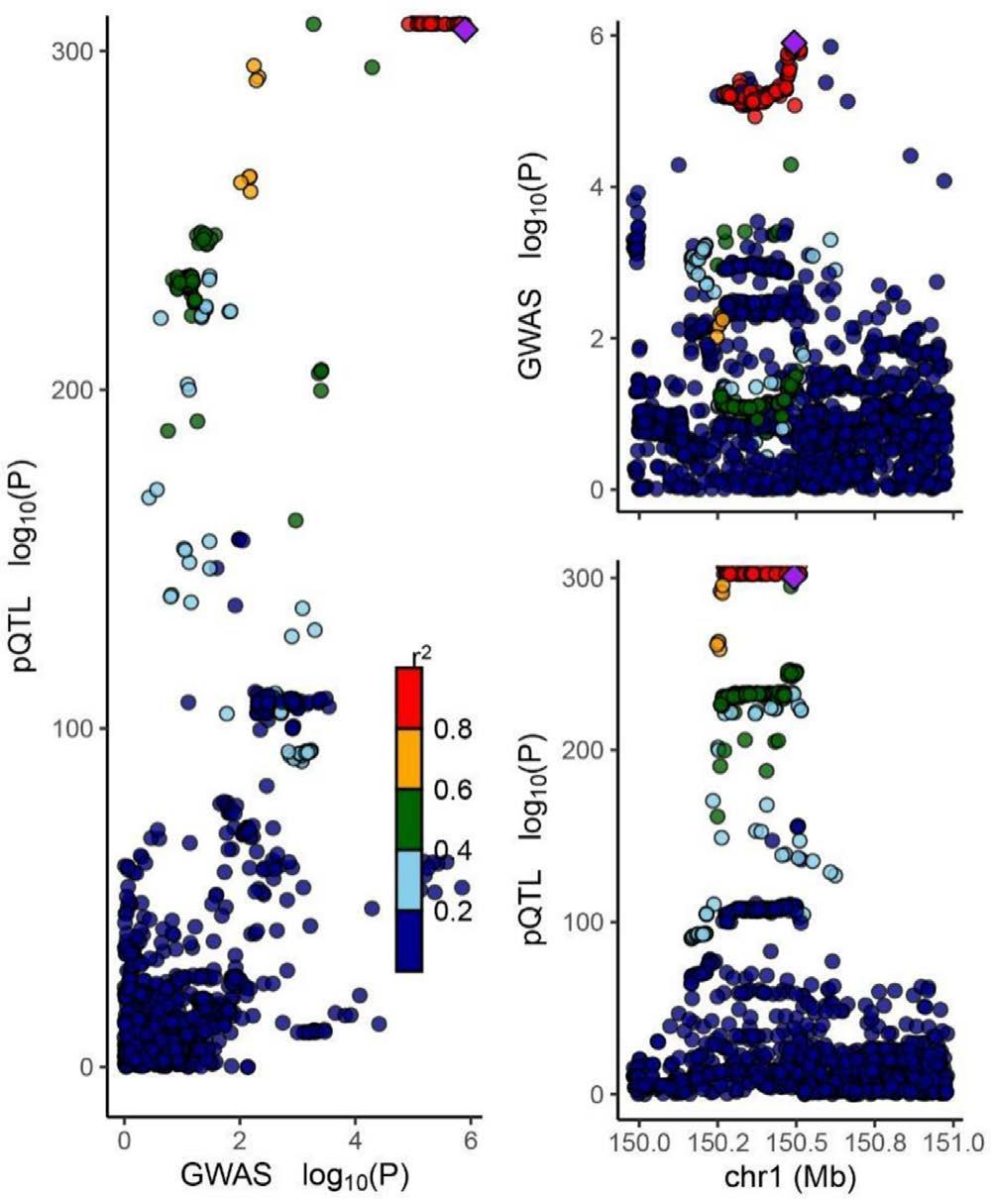
Locus-compare scatter plot for the association signals at ECM1. ECM1 = extracellular matrix protein 1.

Colocalization analysis reports the probability that each protein shares the same variant between the GWAS and pQTL data, referred to as hypothesis 4 (PP4). In the colocalization analysis of plasma GWAS and pQTL related to OA, genetic colocalization evidence based on PP4 > 80% indicated that ECM1 played a significant role in OA risk, with a PP4 value of 0.99 (Fig. 3).

### 3.5. Evaluation of ECM1 gene expression and protein levels in patients with KOA

**Figure 4.**
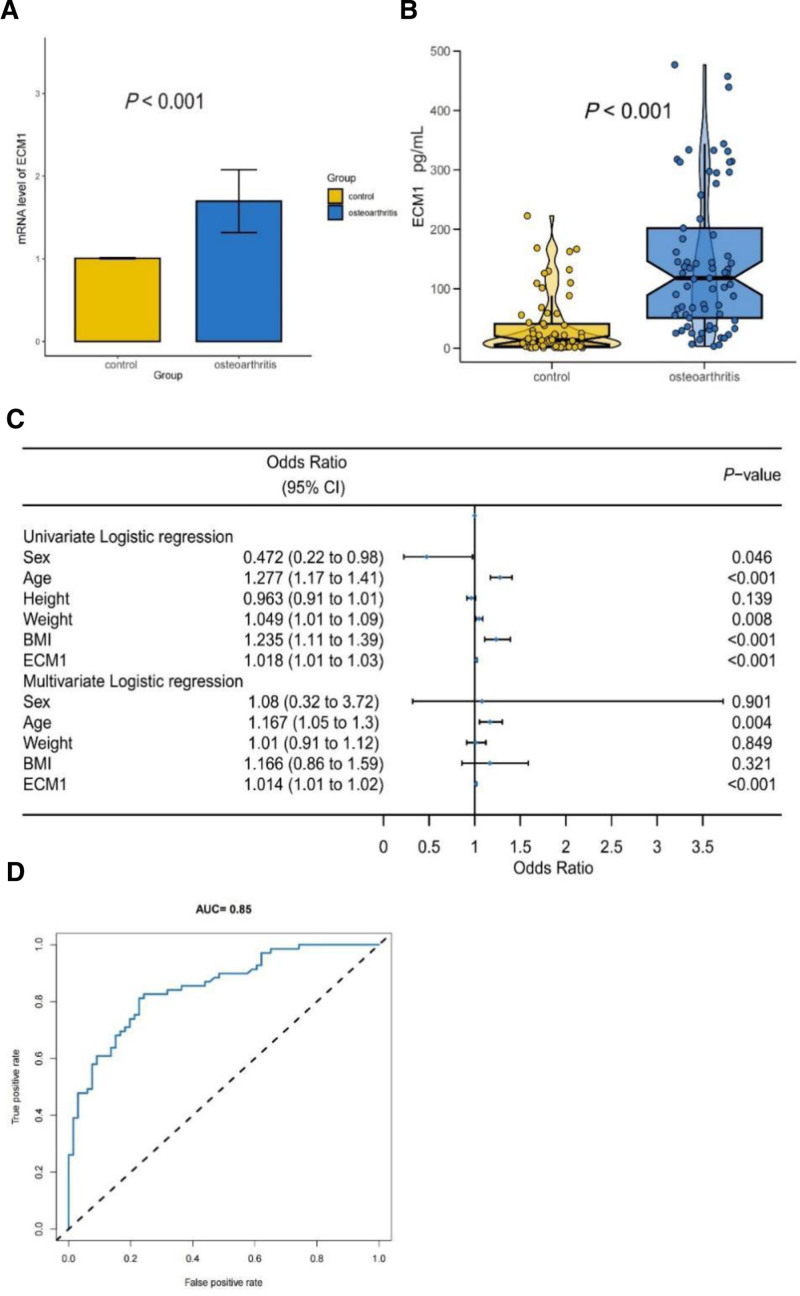
Clinical validation of ECM1 protein levels in blood samples. (A) RT-qPCR analysis results for ECM1. (B) ELISA analysis showing the difference in ECM1 levels between the KOA and control groups. (C) Univariate and multivariate logistic regression analyses. (D) ROC curve for ECM1, demonstrating its diagnostic value. ECM1 = extracellular matrix protein 1, ELISA = enzyme-linked immunosorbent assay, KOA = knee osteoarthritis, ROC = receiver operating characteristic.

The expression level of ECM1 was assessed using RT-qPCR. From November 2023 to April 2024, 64 participants (32 healthy controls and 32 patients with KOA) were successfully enrolled according to the inclusion and exclusion criteria. The basic clinical characteristics of the participants included sex, age, height, weight, and body mass index (BMI) (Table 3). The RT-qPCR results indicated that the mRNA expression level of ECM1 in the KOA group (1.68) increased by 0.68 compared to that in the non-OA group, showing statistically significant difference (Fig. 4A). Subsequently, serum samples were collected for the ELISA measurement of ECM1 protein levels.

**Table 3:** Clinical sample information for RT-PCR.

Variables	Total (n = 64)	Non-KOA (n = 32)	KOA (n = 32)	*P*
Sex, n (%)				.594
Female	43 (67)	23 (72)	20 (62)	
Male	21 (33)	9 (28)	12 (38)	
Age, median (Q1, Q3)	60.5 (56.75, 63.25)	59 (56, 63.25)	61 (57, 63.5)	.247
Height, median (Q1, Q3)	160 (158, 169.25)	160 (159.5, 165.5)	161 (158, 170)	.776
Weight (mean ± SD)	66.18 ± 10.39	63.11 ± 7.6	69.25 ± 11.93	.017
BMI (mean ± SD)	24.96 ± 3.35	23.91 ± 2.43	26.01 ± 3.82	.012

BMI = body mass index, KOA = knee osteoarthritis.

From January 2024 to April 2024, 135 participants (66 healthy controls and 69 patients with KOA) were included according to the same criteria. Basic clinical characteristics such as sex, age, height, weight, and BMI were documented (Table 4). Age, weight, and BMI were significantly different between the 2 groups (*P* < .05). ELISA results indicated that the ECM1 levels in the KOA and control groups were 118 pg/mL and 13.48 pg/mL, respectively, with the difference in ECM1 levels between the 2 groups showing statistical significance (Fig. 4B). Both univariate and multivariate logistic regression analyses were performed to evaluate the diagnostic efficacy of ECM1 expression in relation to the clinical characteristics of KOA (Fig. 4C). Ultimately, age and ECM1 were identified as independent factors influencing diagnosis (*P *< .01), whereas weight and BMI did not show statistically significant effects on diagnostic efficacy in KOA. Furthermore, ROC curve analysis revealed an AUC value of 0.85, suggesting that ECM1 has considerable diagnostic value in KOA (Fig. 4D).

**Table 4:** Basic information of clinical samples used in ELISA.

Variables	Total (n = 135)	Non-KOA (n = 66)	KOA (n = 69)	*P*
Sex, n (%)				.067
Female	91 (67)	39 (59)	52 (75)	
Male	44 (33)	27 (41)	17 (25)	
Age, median (Q1, Q3)	61 (57, 66)	58 (56, 61.75)	65 (61, 67)	<.001
Height, median (Q1, Q3)	160 (160, 169.5)	165 (160, 170)	160 (160, 167)	.164
Weight (mean ± SD)	67.49 ± 10.41	65 ± 8.77	69.87 ± 11.32	.006
BMI (mean ± SD)	25.31 ± 3.57	24.09 ± 2.67	26.48 ± 3.93	<.001

BMI = body mass index, ELISA = enzyme-linked immunosorbent assay, KOA = knee osteoarthritis.

## 4. Discussion

In this study, we found that ECM1 may be an important risk factor for KOA. Through PWAS analysis, we preliminarily identified the potential impact of ECM1 on OA, which was further validated using Gene Expression Omnibus data showing elevated expression levels of ECM1 in OA samples. To corroborate these findings, we utilized ROC curve prediction, yielding an AUC value of 0.7, indicating a significant inclination toward good sensitivity and specificity as a diagnostic marker, thereby supporting the hypothesis that ECM1 is related to OA. Additionally, we performed a COLOC analysis to assess the likelihood that certain SNPs contribute to OA by altering the expression of specific proteins, which enhanced the reliability of our results to some extent. Not only did we conduct theoretical analyses, as mentioned above, but also performed a comparison between patients with KOA and healthy individuals, revealing that ECM1 expression levels were higher in patients with KOA. The AUC value of 0.85 further underscores the diagnostic value of this marker in patients with KOA.

The extracellular matrix (ECM) of articular cartilage forms a 3-dimensional network, primarily composed of interlaced collagen fibers and aggregates of proteoglycans, which play a crucial role in maintaining the structural integrity and mechanical properties of the tissue.^[52]^ This complex architecture is essential for the load-bearing function of the cartilage, as it provides the necessary resilience and support during mechanical loading. The ECM not only serves as a scaffold for chondrocytes but also influences their behavior through biochemical and mechanical signals.^[53–55]^ During the progression of OA, the most common musculoskeletal disease, the ECM undergoes a series of chemical and structural changes that play crucial roles in disease onset and progression of the disease.^[56–58]^ Although the molecular mechanisms involved in the pathological remodeling of ECM are considered essential, they remain incompletely understood. ECM1 is a component of the ECM that may play a significant role in regulating ECM function, influencing cell behavior, and affecting disease progression under specific physiological or pathological conditions. As a key protein in the field of ECM research, ECM1 is vital for understanding the structure and function of the ECM.

ECM1 is a secreted multifunctional glycoprotein involved in regulating various disease processes, including breast cancer,^[59–63]^ asthma,^[64,65]^ diabetic retinopathy,^[66]^ and inflammatory bowel disease.^[67–70]^ Additionally, ECM1 is expressed in bone and cartilage. Increasing evidence has highlighted the crucial role of ECM1 in the regulation of chondrocyte proliferation, hypertrophy, and maturation during endochondral ossification. In OA, ECM1 has been shown to be overexpressed in human articular cartilage, where it interacts with various signaling pathways that regulate cartilage integrity and degradation. For instance, CCN1 (Cyr61), another protein found in osteoarthritic cartilage, has been identified as an inhibitor of ADAMTS-4, an aggrecanase that contributes to cartilage breakdown. This interaction suggests that ECM1 plays a protective role in cartilage by modulating the activity of these enzymes.^[71]^ Furthermore, ECM1 expression is not limited to pathological conditions; it is also essential for normal skeletal development. In zebrafish, the Ucma gene, which is closely related to ECM1, has been shown to be critical for skeletal development. Knockdown of Ucma results in severe growth retardation and skeletal malformations, underscoring the importance of ECM1 and its homologs in the proper formation and maintenance of cartilage and bone.^[72]^ This highlights the potential of ECM1 as a therapeutic target in regenerative medicine, particularly for conditions that affect cartilage and bone health. Recombinant ECM1 inhibited alkaline phosphatase activity and mineralization in vitro in mouse embryonic metatarsal explants. In vivo, ECM1 overexpression delayed cartilage development and endochondral ossification at the growth plate, partly due to reduced PGRN expression. Microarray analyses have identified ECM1 as a late-response gene to parathyroid hormone-related peptides that regulate the rate of chondrocyte proliferation and hypertrophy in the growth plate. Given the critical role of ECM1 in bone development, further investigation into whether ECM1 is involved in the pathological hypertrophy of articular chondrocytes and matrix degradation associated with OA is essential.^[73]^ Research has indicated that cartilage destruction increases the expression of ECM1, thereby exacerbating the progression of OA, in part by reducing PRG4 expression through a TGF-β/PKA/CREB-dependent mechanism.^[74]^ This underscores the importance of ECM1 in OA progression. Targeting ECM1 is a potential therapeutic strategy for the treatment of OA. This finding aligns with the results of our study, which showed a colocalization probability (PP4) of 0.99 ECM1, highlighting its significance in KOA.

This study had certain limitations. First, this was a single-center validation study, which may introduce regional biases, and the generalizability of the results should be interpreted with caution. Secondly, although ECM1 was identified as a risk factor for KOA based on data from European populations, our study was conducted in an Asian cohort. Thus, potential ethnic differences warrant further investigation to elucidate the relationship between ECM1 expression and KOA. Finally, this research primarily focused on the diagnostic value of ECM1 in KOA, lacking a comprehensive exploration of the underlying mechanisms by which ECM1 contributes to the disease process. Future studies should investigate the role of ECM1 in KOA progression and its potential mechanisms to enhance our understanding of its function and impact under these conditions.

## 5. Conclusion

This study conducted PWAS analysis to explore the proteomic mechanisms underlying the pathogenesis of KOA. Among the proteins identified, ECM1 is considered to play a significant role in the development of KOA and holds important value for the further identification of new diagnostic and therapeutic targets for this condition.

## Acknowledgments

The authors wish to acknowledge the patients who were involved in this study.

## Author contributions

**Conceptualization:** Xuelian Yin.

**Data curation:** Huadong Wang.

**Methodology:** Xuelian Yin.

**Software:** Yinhui Yao.

**Validation:** Yinhui Yao.

**Writing – original draft:** Qingzhu Zhang.

**Writing – review & editing:** Qingzhu Zhang.
